# Influenza vaccination in breast cancer patients during subcutaneous trastuzumab in adjuvant setting

**DOI:** 10.1007/s10549-020-05815-y

**Published:** 2020-08-01

**Authors:** Therse Björkin Joona, Evangelos Digkas, Anna-Karin Wennstig, Karin Nyström, Andreas Nearchou, Cecilia Nilsson, Karlis Pauksens, Antonis Valachis

**Affiliations:** 1 Department of Oncology, Västerås Central Hospital, Västerås, Sweden; 2 Department of Oncology, Mälarsjukhuset, Eskilstuna, Sweden; 3 Department of Oncology, Sundsvall Hospital, Sundsvall, Sweden; 4 Department of Oncology, Faculty of Medicine & Health, Örebro University, 70182 Örebro, SE Sweden; 5 Department of Infectious Diseases, Uppsala University Hospital, Uppsala, Sweden

**Keywords:** Influenza, Vaccination, Breast cancer, Immunogenicity, Trastuzumab

## Abstract

**Background:**

Despite the current recommendation for influenza vaccination in cancer patients with active oncological therapy, limited data are available on the efficacy of vaccination in cancer patients receiving targeted therapies. We aimed to investigate the immunogenicity and tolerability of influenza vaccination in breast cancer patients treated with trastuzumab in adjuvant setting.

**Methods:**

A prospective open-label multicenter study was performed including patients with breast cancer during trastuzumab treatment in adjuvant setting and healthy controls. Blood samples were taken before, 4 weeks after, and 12 weeks after a single dose of trivalent influenza vaccine containing inactivated A/California/7/2009 (H1N1) pdm09, A/Hongkong4801/2014 (H3N2), and B/Brisbane/60/2008. Levels of serum antibody titers to hemagglutinin for H1N1 and influenza B strains were measured.

**Results:**

Twenty breast cancer patients and 37 controls were included in the study. No difference in seroprotection rate between trastuzumab-treated patients and controls was observed for either H1N1 (100% in both groups) or B strain (78.9% vs. 89.2%, *p* value = 0.423). A statistically significant increase in geometric mean titers from baseline was seen in both groups and was evident both 4 weeks and 12 weeks after vaccination. Adverse events in the trastuzumab-treated group were uncommon and mild with only one serious adverse event not related to vaccination.

**Conclusion:**

Breast cancer patients treated with trastuzumab in adjuvant setting seem to benefit from influenza vaccination in terms of immunogenicity without increasing the risk for adverse events. The current data support the recommendation to offer influenza vaccination in breast cancer patients treated with this type of targeted therapy.

## Introduction

Patients with cancer are at increased risk for infections and their complications due to immunosuppression caused by the underlying malignant disease or its therapy [1]. Influenza is an acute viral infection of the respiratory tract that causes annual epidemics across the world. In patients with cancer, influenza infection has been reported to prolong the days of hospital stay and increase complications and mortality [2, 3].

Influenza infection in cancer patients during cancer therapy could indirectly result in suboptimal cancer treatment by causing delays in treatment [1]. As a result, yearly influenza vaccination is strongly recommended for patients with cancer receiving chemotherapy or other immune-modulating therapies [4–6] since the development of protective antibodies after vaccination in this population has been proved to be adequate when compared with patients not receiving chemotherapy or healthy controls, without major complications attributable to vaccination [1]. Besides, the clinical effectiveness of influenza vaccination in cancer patients seems to be significant although the strength of current evidence is weak [7].

Trastuzumab (Herceptin®) is a monoclonal antibody that interferes with the growth factor receptor HER2 (which is overexpressed in about 20% of breast cancer patients) and its use have changed the natural history of the HER2-positive breast cancer by improving the disease-free and overall survival [8]. Trastuzumab is now considered as the standard treatment option in for breast cancer patients with HER2-overexpressing breast cancer in all treatment settings namely neoadjuvant, adjuvant and metastatic [9]. In adjuvant setting, trastuzumab is given concurrently with chemotherapy and then as monotherapy for a total of one year of trastuzumab therapy [10, 11].

A part of trastuzumab’s mechanism of action seems to be through the activation of the immune system. Indeed, a growing body of preclinical and clinical evidence shows that the immune system contributes substantially to the therapeutic effects of trastuzumab [12]. Among the several effects of trastuzumab on the immune system, it has been shown to enhance specific mechanisms for better antigen uptake and presentation by dendritic cells [12] which is a basic step on the immunological mechanism of vaccination as well [13].

Despite the recommendation for universal influenza vaccination in all cancer patients with active treatment, including breast cancer patients during adjuvant therapy, there are no preclinical or clinical evidence on the efficacy of vaccination in patients treated with trastuzumab. Considering the stimulation of immune system caused by trastuzumab, one could argue that those patients would achieve higher immunological response to vaccine than individuals without trastuzumab therapy. However, a negative effect of trastuzumab on immune response after influenza vaccination cannot be totally excluded since the pathways of interactions between trastuzumab and immune system are not fully understood.

The aim of the present study was to investigate the immunogenicity of influenza vaccination in breast cancer patients treated with trastuzumab in adjuvant setting and explore the tolerability of influenza vaccination to this group of breast cancer patients.

## Methods

### Study design and patient selection

This prospective open-label study was conducted in four hospitals in Sweden (Mälarsjukhuset, Sundsvall General Hospital, Västerås General Hospital, Örebro University Hospital) during the 2016–2017 winter season. Female patients > 18 years of age with stage I, II, or operable stage III HER2-positive breast cancer who had undergone breast cancer surgery, completed their neoadjuvant or adjuvant chemotherapy, and were under treatment with adjuvant trastuzumab monotherapy were eligible for the study.

As control group we used healthy employees of two of the participating hospitals (Mälarsjukhuset, Västerås General Hospital) that voluntarily participate in the yearly influenza vaccination campaign for health care personnel.

The HER2-positivity was defined as amplification verified by FISH or CISH or 3+ in immunohistochemistry. The breast cancer surgery method could be either breast-conserving or mastectomy, and no clinical or radiological signs of distant metastases were suspected. Local or locoregional radiotherapy prior to the study inclusion was allowed, as was ongoing endocrine therapy with tamoxifen or aromatase inhibitors. Chemotherapy use prior to study entry was mandatory for all the eligible patients. The time between the last cycle of chemotherapy and inclusion to the study had to be at least one month. The patients were between cycle 2 and 13 (of 14 in total) in their trastuzumab monotherapy phase, receiving trastuzumab either intravenously or subcutaneously.

We excluded patients with inflammatory breast cancer or metastatic disease, those with known allergy to any of the vaccine components, patients treated with steroids (> 15 mg prednisolone or equivalent daily) or other immunosuppressive agents or immunosuppressive disease, and patients with a risk for a possibly change of anti-cancer treatment before the first evaluation of immunogenicity following the first injection (to avoid bias related to any therapy changes).

### Vaccination

All patients and healthy controls were vaccinated intramuscularly with a single dose of the inactivated trivalent non-adjuvant seasonal influenza vaccine Vaxigrip (Sanofi Pasteur MSD). The vaccine licensed for the 2016–2017 season contained an A/California/7/2009 (H1N1) pdm09-like virus, an A/Hongkong4801/2014 (H3N2)-like virus, and a B/Brisbane/60/2008-like virus.

In patients treated with trastuzumab, the vaccine was administered 14 ± 2 days after trastuzumab dose, extrapolating the recommendation on the preferred vaccination time during chemotherapy, namely at mid-cycle, preferably 2 weeks after chemotherapy [4].

Participants were observed for 15 min after vaccination to capture any immediate adverse reactions.

### Measurement of antibody titers

Blood samples were drawn before the vaccination, on day 28 ± 3 and on day 90 ± 7 after vaccination for assessment of hemagglutination-inhibition (HI) for H1N1 and influenza B strain using a standard method. All serum specimens were kept at ‒70 °C until analysis. All samples were assayed at the Public Health Agency of Sweden.

### Outcomes of interest

The primary outcome of the study was the immunogenicity to influenza vaccination in breast cancer patients treated with adjuvant trastuzumab compared to healthy controls expressed as post-vaccination seroconversion rate (SCR) defined as a variable-fold increase in HI titer as a function of prevaccination titer at day 28 ± 3, an approach that seems to derive more plausible estimate of SCR in case of high background immunity [14]

Secondary outcomes were SCR at day 90 ± 7, seroprotection rate (SPR), defined as the percentage of patients with a titer of least 1:40, geometric mean titers (GMT) of HI after vaccination compared to baseline, and tolerability of influenza vaccination during trastuzumab treatment.

### Safety surveillance

The safety of the influenza vaccination in patients treated with trastuzumab and in controls was evaluated by using a questionnaire at baseline and then on day 7, 8, and 21. Adverse events (AEs) due to vaccination were graded according to the Food and Drug Association’s (FDA) guidance for toxicity grading scale for healthy adults enrolled in preventive vaccine clinical trials [15].

### Statistical analyses

Categorical variables were described as frequencies and percentages, and continuous variables as medians and interquartile range (IQR). The comparisons of SCR and SPR between patients and healthy controls were performed with Chi-square test or Fisher´s exact test whereas the changes on GMT from baseline in each group was performed with mixed linear models.

Considering the lower antibody response to influenza vaccination in the elderly population [16], we performed multiple logistic regression analyses for SCR and SPR in the two different time points (28 ± 3 days and 90 ± 7 after vaccination) in which we included age (< 65 years old vs. ≥ 65 years old), prior influenza vaccination, and study group (patients treated with trastuzumab vs. healthy controls) as covariates.

A “non-inferiority trial” design was applied to calculate the sample size on this study according to Rao et al. [17]. Assuming the SCR one month after vaccination for each strain (primary outcome) to be 70% for healthy controls and 65% for trastuzumab-treated patients and the non-inferiority margin regarding the SCR difference between the two groups to be −10%, the non-inferiority would be achieved if the lower limit of the 95% confidence interval (CI) of the SCR difference between the groups was greater than −15%. Based on these assumptions, 57 study participants (1:2 allocation ratio; 19 in the trastuzumab-treated group and 38 healthy controls) were required to have 80% power (*α* = 0.05) to prove a non-inferiority regarding SCR between the two groups.

Statistical analyses were performed with the statistical software SPSS (version 24.0; SPSS Inc., Chicago, Ill., USA)

### Ethics

Written informed consent was obtained from each patient and healthy control. The study protocol was approved by the Research Ethics Committee in Stockholm and the Swedish Medical Products Agency and was performed in accordance with the Declaration of Helsinki and its later amendments, the Good Clinical Practice guidelines, and the International Council for Harmonisation of Technical Requirements for Pharmaceuticals for Human Use (ICH) regulatory guidelines.

## Results

### Study cohort

During the inclusion period, 20 breast cancer patients treated with trastuzumab and 38 healthy controls were enrolled. One healthy control withdrew the informed consent after the baseline blood sampling and, therefore, excluded from the analysis. All patients and healthy controls except the one who withdrew the informed consent completed the study with blood samples at baseline and at the two predefined time points after vaccination. All blood samples could be analyzed except from one patient whose blood sample at 3 months after vaccination could not be processed and was excluded from the immunogenicity analysis.

The participants’ baseline demographic and clinical characteristics are summarized in Table 1. The median age in the trastuzumab-treated patients was 59.5 years compared to 49 years old in healthy controls. Most of the participants in both groups (70% in trastuzumab-treated patients; 89% in healthy controls) have previously been vaccinated with influenza vaccine. All patients received trastuzumab as subcutaneous formulation whereas the median number of trastuzumab cycles before vaccination was 6.5. All patients received chemotherapy with a medina time between end of chemotherapy and influenza vaccination 6 months (IQR: 3–8.5 months).

**Table 1:** Characteristics of study cohort and controls

Variable	Trastuzumab-treated patients (*n* = 20)	Healthy controls (*n* = 37)
Age, median (IQR)	59.5 (54.0–69.5)	49 (42.0–54.5)
BMI	24.7 (22.7–29.9)	25.7 (22.5–29.2)
Previous influenza vaccination	14 (70)	33 (89)
Previous influenza infection	1 (5)	11 (30)
Baseline geometric mean titers (SD)		
B	16.39 (2.09)	34.43 (3.11)
HINI	28.33 (2.82)	102.07 (2.56)
Baseline seroprotection		
B	4 (20)	21 (57)
HINI	9 (45)	34 (92)
Tumor characteristics		
T size in mm, median (IQR)	17.5 (12.0–23.8)	NA
N0	16 (80)	
ER-positive	13 (65)	
PgR-positive	9 (45)	
Grade 3	8 (40)	
Ki-67, median (IQR)	29.5 (17.0–67.3)	
Treatment characteristics		
Breast-conserving surgery	14 (70)	NA
Sentinel lymph node biopsy	15 (75)	
Neoadjuvant chemotherapy	2 (10)	
Postoperative radiotherapy	17 (85)	
Endocrine therapy	14 (70)	
sc Trastuzumab formulation	20 (100)	
Trastuzumab monotherapy cycles before influenza vaccination, median (IQR)	6.5 (4.0–8.8)	

*IQR* interquartile range, *SD* standard deviation, *T* tumor, *ER* estrogen receptor, *PgR* progesterone receptor, *sc* subcutaneous, *NA* not applicable

### Seroconversion and seroprotection rates

The SCR and SPR in trastuzumab-treated patients and healthy controls are shown in Table 2.

**Table 2:** Seroconversion and seroprotection rates after vaccination in trastuzumab-treated patients and healthy controls

Variable	Type of influenza	Trastuzumab-treated patients (*n* = 20)	Healthy controls(*n* = 37)	*P* value
Seroconversion at 4 weeks	B	13 (65.0)	24 (64.9)	1.000
	HINI	14 (70.0)	23 (62.2)	0.554
Seroconversion at 3 months	B	11 (57.9)*	19 (51.4)	0.779
	HINI	14 (77.8)*	16 (43.2)	0.022
Seroprotection at 4 weeks	B	18 (90)	37 (100)	0.119
	HINI	18 (90)	37 (100)	0.339
Seroprotection at 3 months	B	15 (78.9)*	33 (89.2)	0.423
	HINI	19 (100)*	37 (100)	NC

*NC* not calculated

*Denominator for seroconversion and seroprotection rates at 3 months in the trastuzumab-treated patients is 19 because there was one patient whose specimen could not be processed

At baseline, 92% of healthy controls had seroprotective antibody titers for H1N1 strain compared to 45% in the trastuzumab-treated patients whereas the seroprotection for influenza B strain was 57% for healthy controls compared to 20% for trastuzumab-treated patients.

SCR at 4 weeks, as calculated according to Chandramohan et al. [14] to adjust for high level of antibody titers at baseline, was similar between the trastuzumab-treated patients and healthy controls for both influenza B and H1N1 strains (65.0% vs. 64.9% for influenza B, Δ = 0.1%, 95% CI –14.3 to 13.4%; 70.0% vs. 62.2% for H1N1, Δ = 7.8%, 95% CI −12.1% to 13.1%). SCR at 3 months was also similar for influenza B (57.9% vs. 51.1%), but somewhat higher in favor of trastuzumab-treated patients for H1N1 (77.8% vs. 43.2%).

Trastuzumab-treated group was not associated with either higher or lower SCR in multiple logistic regression models (adjusted for age and prior influenza vaccination) that performed separately for each strain and each time point. The only factor that was independently associated with SCR was age where patients < 65 years old had a higher SCR for influenza B strain at 3 months (Odds ratio (OR): 23.3; 95% Confidence Interval (CI) 2.1–254.1, *p* value = 0.01) but not for H1N1 strain at the same time point (OR: 2.2; 95% CI 2.7–18.3, *p* value = 0.452).

SPR was comparable between trastuzumab-treated patients and healthy controls for both influenza B and H1N1 strains at 4 weeks and 3 months, respectively (Table 2).

When SCR and SPR in the trastuzumab-treated group were stratified based on age (< 65 years old vs. ≥ 65 years old), similar SCR were observed for H1N1 (at 4 weeks: 66.7% vs. 75%; at 3 months: 66.7% vs. 75%) and influenza B strain at 4 weeks (83.3% vs. 62.5%) but not for B strain at 3 months (75% vs. 25%) whereas SPR was comparable in both strains and time periods (H1N1 at 4 weeks: 100% vs. 100%; H1N1 at 3 months: 83.3% vs. 100%; B strain at 4 weeks: 100% vs. 75%; B strain at 3 months: 83.3% vs. 62.5%).

### Serologic response according to geometric mean titers

Prevaccination GMTs were higher in healthy controls compared to trastuzumab-treated patients for both strains.

**Fig. 1:**
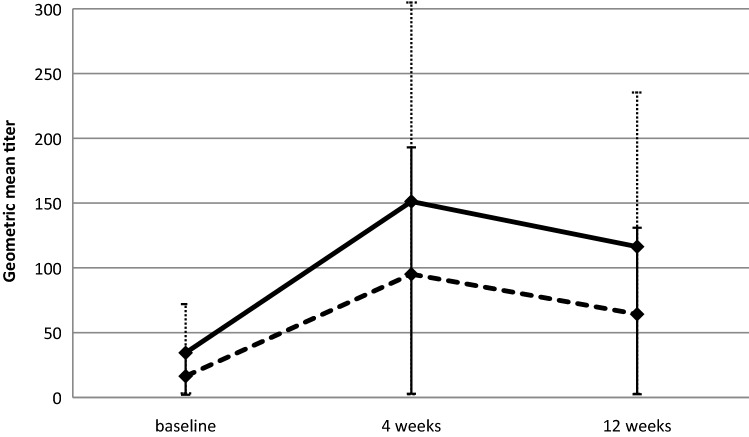
Serologic response to influenza vaccine against influenza B expressed as geometric mean titers for trastuzumab-treated patients (dashed line) and healthy controls (solid line)

Immunogenicity analysis for the influenza B strain (Fig. 1) using repeated measures ANOVA showed that there were significant differences among the 3 time points in both trastuzumab-treated patients (baseline vs. 4 weeks *p* value < 0.001; baseline vs. 12 weeks *p* value = 0.042) and healthy controls (baseline vs. 4 weeks *p* value < 0.001; baseline vs. 12 weeks *p* value = 0.012).

**Fig. 2:**
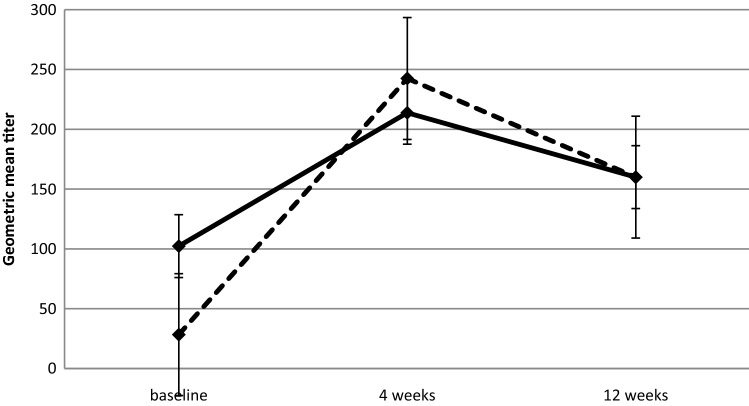
Serologic response to influenza vaccine against H1N1 as geometric mean titers for trastuzumab-treated patients (dashed line) and healthy controls (solid line)

Similarly, immunogenicity analysis for the H1N1 strain (Fig. 2) showed that there were significant differences among the 3 time points in both trastuzumab-treated patients (baseline vs. 4 weeks *p* value < 0.001; baseline vs. 12 weeks *p* value = 0.039) and healthy controls (baseline vs. 4 weeks *p* value < 0.001; baseline vs. 12 weeks *p* value = 0.014)

### Adverse events

Five patients (25%) in the trastuzumab-treated group experienced AEs in the 21 days following vaccination including two patients with local pain, one with arthralgia, one with chills, and one with myalgia. All AEs were reported within one week from vaccination, were mild, and resolved spontaneously within 1–2 days. No patients reported influenza or influenza-like illness during the study period. One serious AE was recorded in a patient with skin and soft tissue infection at the surgical site on the breast that resolved with intravenous antibiotics and assessed as not related to vaccination.

## Discussion

The results of this prospective study suggest an adequate immune response to influenza vaccination among breast cancer patients treated with adjuvant trastuzumab which seems to be comparable to healthy controls without an increased risk for adverse events.

Although the impact of conventional systemic cancer treatment strategies as chemotherapy on vaccine immunogenicity in patients with solid tumors is extensively studied [1, 7], few studies have specifically investigated the potential impact of targeted therapies, as multi-kinase inhibitors and monoclonal antibodies, on vaccine immunogenicity. In the VACANCE study, an adequate immune response to vaccination against H1N1 strain was observed in nine patients treated with different targeted therapies, including three patients with trastuzumab [18]. Correspondingly, Xu et al. found comparable seroconversion rate after vaccination against H1N1 strain between eight patients treated with non-myelosuppressive targeted therapies and 44 healthy controls [19]. Mulder et al. found similar seroprotection rates to seasonal influenza vaccination between 22 patients treated with multi-kinase inhibitors sunitinib or sorafenib and 11 healthy controls [20]. Our results add to the current limited evidence supporting the adequate immunogenicity of influenza vaccination in patients with solid tumors treated with targeted therapies.

The higher SCR for H1N1 strain at three months in favor of trastuzumab-treated patients could be mainly explained by the higher levels of antibody titers at baseline in healthy controls rather than by a true difference with a biologic rationale. In fact, this difference was not evident in multivariate analysis using prior influenza vaccination as covariate. Considering the differences in baseline characteristics between patients and healthy controls, one could argue that choosing breast cancer patients not receiving chemotherapy and trastuzumab as control group would be more appropriate. This approach

The association between age and SCR observed in this study, namely the risk for lower SCR in elderly, is in line with well-founded evidence [16] underlying the need for more immunogenic vaccine strategies for the elderly.

Several limitations of the study should be considered when interpreting the results. First, the number of patients in the trastuzumab-treated group is relatively low. Despite the limited number of patients, we reached the planned study sample size according to the “non-inferiority trial” design as it describes above. Second, the level of antibody titers at baseline was high, especially for the healthy controls, which can lead to underestimation of SCR. To limit this risk, we used the Chandramohan et al. approach [14] to calculate SCR for both groups considering the prevaccination antibody titers in the estimates. Another potential limitation is that we included patients treated with trastuzumab in adjuvant setting and, as a result, the generalizability of the results in patients treated with dual HER2-blockade or those with metastatic breast cancer treated only with trastuzumab might be considered cautiously. In addition, all patients received the subcutaneous formulation of trastuzumab. Although intravenous and subcutaneous formulations of trastuzumab have similar immunogenicity, efficacy, and tolerability [21], a potential difference on how the formulations can influence the immunogenicity of influenza vaccination cannot be completely excluded. Finally, the study aimed to investigate immunogenicity, thus no data on the clinical efficacy of influenza vaccination in trastuzumab-treated patients have been collected.

Despite these caveats, this study offers additional evidence on the clinical-relevant question on whether cancer patients treated with monoclonal antibodies as trastuzumab can derive benefit from influenza vaccination. Based on our results, breast cancer patients treated with trastuzumab in adjuvant setting seem to benefit from influenza vaccination in terms of immunogenicity without increasing the risk for adverse events. Consequently, our data support the recommendation to offer influenza vaccination in breast cancer patients during trastuzumab treatment.

## References

[1] Pollyea D, Brown JMY, Horning SJ (2010). Utility of influenza vaccination for oncology patients. J Clin Oncol.

[2] Tai Y, Lee TC, Chang HL, Chen KT (2009). Epidemiology and outcomes of hospitalization of influenza in the cancer population in Taiwan. J Cancer Res Clin Oncol.

[3] Cooksley CD, Avritscher EB, Bekele BN, Rolston KV, Geraci JM, Elting LS (2005). Epidemiology and outcomes of serious influenza-related infections in the cancer population. Cancer.

[4] Rubin LG, Levin MJ, Ljungman P, Davies EG, Avery R, Tomblyn M (2014). 2013 IDSA clinical practice guidelines for vaccination in immunocompromised host. Clin Infect Dis.

[5] Pedrazolli P, Baldanti F, Donatelli I, Castrucci MR, Puglisi F, Silvestris N (2014). Vaccination of seasonal influenza in patients with cancer: recommendations of the Italian society of medical oncology (AIMO). Ann Oncol.

[6] Recommendations of the Public Health Agency of Sweden about influenza vaccination. https://www.folkhalsomyndigheten.se/smittskydd-beredskap/vaccinationer/vacciner-a-o/influensa/. Accessed: 11 Feb 2020.

[7] Bitterman R, Eliakim-Raz N, Vinograd I, Zalmanovici Trestioreanu A, Leibovici L, Paul M (2018). Influenza vaccines in immunosuppressed adults with cancer. Cochrane Database Syst Rev.

[8] Dawood S, Broglio K, Buzdar AU, Hortobagyi GN, Gjordano SH (2010). Prognosis of women with metastatic breast cancer by HER2 status and trastuzumab treatment: an institutionalbased review. J Clin Oncol.

[9] Hudis CA (2007). Trastuzumab – mechanism of action and use in clinical practice. N Engl J Med.

[10] Cameron D, Piccart-Gebhart MJ, Gelber RD, Procter M, Goldhirsch A, de Azambuja E, Herceptin Adjuvant (HERA) Trial Study Team (2017). 11 years follow-up of trastuzumab after adjuvant chemotherapy in HER2-positive early breast cancer: final analysis of the HERceptin adjuvant (HERA) trial. Lancet.

[11] Romond EH, Perez EA, Bryant J, Suman VJ, Geyer CE, Davidson NE (2005). Trastuzumab plus adjuvant chemotherapy for operable HER2-positive breast cancer. N Engl J Med.

[12] Bianchini G, Gianni L (2014). The immune system and response to HER2-targeted treatment in breast cancer. Lancet Oncol.

[13] Pulendran B, Ahmed R (2011). Immunological mechanisms of vaccination. Nat Immunol.

[14] Chandramohan D, Coleman P, Nelson C, Greenwood B (2007). A new approach to the definition of seroconversion following vaccination in a population with high background antibody concentrations. Vaccine.

[15] FDA Guidance Document. Toxicity grading scale for healthy adult and adolescent volunteers enrolled in preventive vaccine clinical trials. September 2007. https://www.fda.gov/regulatory-information/search-fda-guidance-documents/toxicity-grading-scale-healthy-adult-and-adolescent-volunteers-enrolled-preventive-vaccine-clinical. Accessed Feb 2020. 10.1016/j.vaccine.2023.07.072 37532612

[16] Goodwin K, Viboud C, Simonsen L (2006). Antibody response to influenza vaccination in elderly: a quantitative review. Vaccine.

[17] Rao MR, Blackwelder WC, Troendle JF, Naficy AB, Clemens JD (2002). Sample size determination for phase II studies of new vaccines. Vaccine.

[18] Rousseau B, Loulergue P, Mir O, Krivine A, Kotti S, Viel E (2012). Immunogenicity and safety of the influenza A H1N1v 2009 vaccine in cancer patients treated with cytotoxic chemotherapy and/or targeted therapy: the VACANCE study. Ann Oncol.

[19] Mulder SF, Jacobs JF, Olde Nordkamp MA, Galama JM, Desar IM (2011). Cancer patients treated with sunitinib or sorafenib have sufficient antibody and cellular immune responses to warrant influenza vaccination. Clin Cancer Res.

[20] Xu Y, Methuku N, Coimbatore P, Fitzgerald T, Huang Y, Xiao YY (2012). Immunogenicity of an inactivated monovalent 2009 influenza A (H1N1) vaccine in patients who have cancer. Oncologist.

[21] Jackisch C, Kim SB, Semiglazov V, Melichar B, Pivot X, Hillenbach C (2015). Subcutaneous versus intravenous formulation of trastuzumab for HER2-positive early breast cancer: updated results from the phase III HannaH study. Ann Oncol.

